# Evidence of Long Term Benefit of Morbidity Reduction Due to Praziquantel Treatment Against *Schistosoma Mansoni* in Kigungu Fishing Village in Entebbe, Uganda

**DOI:** 10.4314/ajid.v5i2.66511

**Published:** 2011

**Authors:** Emmanuel I Odongo-Aginya, FK Kironde, MI Lyazi, Harman Sempewo, Rodrigo Correa Oliveira

**Affiliations:** 1 Microbiology Department Gulu University P.O.Box 166 Gulu UGANDA; 2 Faculty of Science, Biochemistry Department, Makerere University Kampala P.O.Box 7062 Kampala UGANDA; 3 Statistician and head of the statistic Child Health Department in Mulago-Makerere. UGANDA; 4 Nsambya Missionary Hospital Kampala P.O.Box Kampala UGANDA; 5 Centro de Rene, Rachou-Fiocruz Laboratorio de Immologia Cellular e Molecular, Av.Agusto de Lima, 1715 Barr Preto, CEP 30190-002 Belo Horizonte, Minas Gerais Brazil

## Abstract

Praziquantel (PZQ) is efficacious against *Schistosoma mansoni*. This was prospective cohort study. This study was carried out at Kigungu fishing village, Entebbe, Uganda. The goal of the study was to establish cost effective regiment for mass drug administration (MDA) of Praziquentel in the morbidity reduction of *S.mansoni* infection. In January 2004, nine hundred and forty five (945) participants were registered in this study. Our analysis was based on examining microscopically three slides prepared from each of 945 stool specimens delivered by each of the participant using modified Kato/Katz method. These included male and female, children and adults living in Kigungu fishing village in Entebbe Uganda. In total 901, cohorts were re-examined for infections clearance six months later in July 2004 and 18 months later in June 2005, 625 cohorts were again re-evaluated for *S.mansoni* infections after the baseline study. At baseline, (448) of 945 (47.5%) cohorts were *S. mansoni* positive. All these participants were treatment with a single oral dose of praziquantel at 40mg/kg. At the same time, 495 (52.5%) were *S. mansoni* negative. Of the 625 (66.3%) cohorts who came back for final review, 80 (12.8%) were still positive for *S. mansoni* while 210 (33.6%) remained negative after the base line treatment with praziquantel. On the other hand 103 (16.3%) of cohorts who were initially negative at the base line became *S.mansoni* positive after 18 months and 213(34.1%) remained negative for *S.mansoni*. The force of re-infection after six months was significant {(P=0.0001), (OR 0.47) CI at 95% (0.31–0.71)}. Nevertheless the force of reinfection was not significant after 18 months {(P=0.766), (OR 0.95) CI at 95% (0.68–1.34)}.The geometric mean eggs excretion of the 80 cohorts who were *S.mansoni* positive at 18 months was 151.967.This did not reach the geometric mean egg excreted by the same cohorts at baseline which was 285.05. The egg excretion was reduced by 46.8%. Similarly there was marked decrease in clinical symptoms amongst the cohorts. Our study suggests evidence of long-term benefit of praziquantel in Kigungu and that a yearly administration of praziquantel to the community could be a regiment for mass drug administration (MAD) for this community to control schistosomiasis morbidity.

## Introduction

Praziquantel (PZQ) is efficacious against *Schistosoma mansoni* (Sleigh et al., 1985). Presently in Uganda, one of schistosomiasis endemic countries in sub Saharan Africa, PZQ is the drug of choice in controlling morbidity due to schistosomiasis because the mean cost of treatment per dose per person in Uganda is about $0.3 (Mott, 1982; Doenhoff et al., 2002). In spite of its relative low cost, Uganda Ministry of Health budget is still unable to procure adequate PZQ for short period mass chemotherapy. Previous studies in the seventies showed that in Uganda, the efficacy of PZQ was evaluated after six months to determine the cure proportion (Odongo-Aginya and Mugisha, 1987). Nevertheless in other tropical countries, few studies evaluated the efficacy of PZQ at the interval of one year (Stelma et al., 1995; Correa-Oliveira, et al., 2000; Davis, 1993). At present, studies in different endemic settings using the single oral dose regiment of 40 (mg/kg wt) of PZQ against *S. mansoni*, and mixed infections, the efficacy of PZQ stands at 60-90 % (Cioli and Pica- Mattoccia, 2000). Extensive use of PZQ in Uganda and elsewhere in the tropics has been linked to the development of parasite resistance to the drug. This is evidenced by reduced cure proportion in humans treated with PZQ in the Richard Toll area of Senegal (Stelma et al., 1995). However, factors like intensity of infections and high transmission of infections have been known to influence schistosomicidal activity of PZQ (Gryseels, 1994). In spite this fact, repeated treatment with PZQ has been shown to improve cure proportion as observed in West Nile districts of Uganda areas of high transmission and intensity (Ongom and Bradley, 1972).

Consequently it was deemed necessary to establish a treatment regimen for schistosomiasis endemic foci of Uganda. This study investigated specifically the effect of PZQ on cure proportion as measured by *S. mansoni* egg reduction in the cohorts 18 months after treatment in Kigungu-fishing village, a community with high exposure risk to *S. mansoni* infection in order to establish morbidity reduction, hence, the patients benefit from treatment with PZQ and cost effective treatment regimen that could be adopted in Uganda.

## Patients and Methods

### Study sample

The participants were registered in the three studies using their study code numbers, names, sex, age, home locations and the names of head of the families. Nine hundred and forty five residents of Kigungu fishing village were registered in this study in January 2004. Their stool specimens were examined microscopically for *S.mansoni* eggs. Out of the 945 participants examined 448 (47.4%) of them were stool positive for *S. mansoni*, and 495 (52.6%) participants were stool negative for *S. mansoni*. Treatment with PZQ at 40mg/kg was administered to the 448 *S. mansoni* positive participants. While the *S.mansoni* stools negative participants were treated for other ailments where necessary. The two categories of participants were requested to report for review in July 2004. Six months later, 901 out of 945 (95.5%) cohorts were reviewed. These consisted of 433 out of 448 (96.7 %) treated *S. mansoni* positive cohorts. On re-examination of their stool specimens, 40 cohorts still had *S.mansoni* egg. They were again treated but were left out of the study. Similarly 468 out 495 (94.5%) who were *S.mansoni* negative at baseline were reviewed. Ninety two of them were found with eggs of *S. mansoni* in their stool. They were treated with PZQ 40mg/kg body weight and left out of the study. Twelve months later 625 out of 769 (81.3%) of the cohorts came back for the third evaluation. Among the 310 cohorts who were stool negative after PZQ clearance at baseline 80 became positive for *S. mansoni* while 230 were still stool negative for *S. mansoni*. Meanwhile among the 315 cohorts who were *S.mansoni* negative at baseline, 102 Patients became infected with *S. mansoni* while 213 cohorts continued to be stool negative for *S. mansoni* (see flow chart below).

### Study area

This study was conducted in Kigungu fishing village, situated along Lake Victoria in Entebbe peninsula. This village is located to the extreme end of the peninsula, at latitude 35° to 38° East and 03° to 07° North. Kigungu is about 15 kilometers from Entebbe Town Municipality and about half a kilometer from the Entebbe international airport. This fishing village was selected because previously studies on *S. mansoni* and other soil-transmitted helminths showed that the village is a focus for these parasites (Odongo-Aginya and Mugisha, 1987). The population of Kigungu is estimated to be 6,000 people with nearly an equal sex 1:1 ratio. They are mainly fishermen and women. Besides fishing, they do a little subsistence farming mostly for food crops. Their water exposure is high, hence, the source of infection and reinfection.

### Procedure of the study

Informed consent was obtained from all the participants. One thousand Residents who had consented to participate in the study and children between 5 and 18 years old who had been granted permission to participate in the study by their parents/guardians and have not taken antischistosomal treatment six months prior to the baseline study were recruited. This explains the reason for the 55 people who were excluded from the base line registration. Those giving their consent and were literate, were asked to sign an official form showing acceptance. Meanwhile the illiterate patients used thumb prints on official form showing acceptance. Patients unwilling to participate in the study were not penalised in any way and normal clinical services and treatments including antischistosomal therapy were not conditioned to the patient's participation in this research.

Residents reported for the study at our outreach clinic in Kigungu primary school between 9 a.m and 12 p.m every project workday. On each working day, thirty consecutive patients in a row, and who met the inclusion criteria, were registered into the study. This was to allow the laboratory technologist to examine the specimens in the afternoon and deliver the stool results the following day in the morning for treatment. The patients to be recruited were interviewed and examined by a physician and a nurse during initial screening. The physicians examined the patients clinically with special attention to condition of the abdominal organs commonly affected by *S. mansoni* worm. Anaemia and fever were also clinically noted. Patients with body temperature greater than 37.5°C had blood smear test for malaria parasites done.

All patients infected with *S. mansoni* were treated at the study site with praziquantel (from Medochemie Ltd.Limassol-Cyprus Europe) at 40 mg per kg body weight. Illnesses, other than schistosomiasis, detected during examination were appropriately treated or referred to other health facilities. Patient's privacy was duly respected.

### Determination of intensity of intestinal worms

A stool container labelled with individual identification was given to each patient to return with about 5–10 gram of stool specimen. Eggs in the stool were quantified using modified Kato-Katz method (Odongo-Aginya, et al., 1997; Mahdi, et al., 1999). Essentially each stool specimen was initially strained through a stainless steel sieve 250µm mesh size to remove artefacts. The strained stool was then used to fill a hole in a template measuring 41.7 milligram of stool. Three separate aliquots of such measured weights were delivered on three separate slides from each stool specimen. About 10µl drop of compound stain consisting of eosin 5% in 10% formalin and nigrosin 7.5% in 10% formalin was added to stool smear on each slide. The stain was stirred in the stool smear on the slide. A wettable cellophane cover slip cut 32 × 41 mm pre soaked in 50% glycerine was placed on the stained stool smear and pressed down. The excess stain from the smear on slide was blotted out on an absorbent paper before the prepared slide was read immediately using objective × 10. The arithmetic mean of the eggs counted in three slides was recorded as the count in 41.7 milligram of stool. To convert the mean egg count into egg per gram of faeces a factor of 24 was multiplied by the mean of the eggs counted i.e. number of eggs (n) × 1000 mg/ 41.7 mg = 24 × n = eggs per gram faeces. Intensities of infections were classified as follows: low 1–100 eggs per gram, medium 101–400 and high ≥ 401eggs per gram of stool (Sleigh et al., 1985).

### Data management and statistical analysis

Data were double entered using Microsoft Excel and crosschecked by different individuals. The arithmetic mean egg counts of the individuals were categorised according to infection intensities as follows 0–100; 101–200; 201–300; 301–400; and ≥ 401. Similarly age group was also categorised into five groups 5–10; 11–20; 21–30; 31–40 and ≥ 41. The cure proportion was calculated from individuals who had no *S. mansoni* egg in their stool after treatment divided by the total number of individuals who had *S. mansoni* egg in their stool before treatment multiply by 100%. The percentage of egg count reduction was calculated as the geometric mean egg count after treatment/ by geometric mean egg count before treatment multiplied by 100%. The T-test was used to compare the intensity of infections between *S. mansoni* amongst 80 patients who were reinfected 18 months later and 102 patients who were *S. mansoni* negative but became *S. mansoni* positive 18 months later at 95% level of confidence while the *Chi* χ^2^ was used to show the force of infection which was evaluated by egg count reduction and the cure proportion after 6 months and 18 months against force of reinfection in six months and 18 months after treatment at 95% level of confidence.

## Results

**Figure 1:**
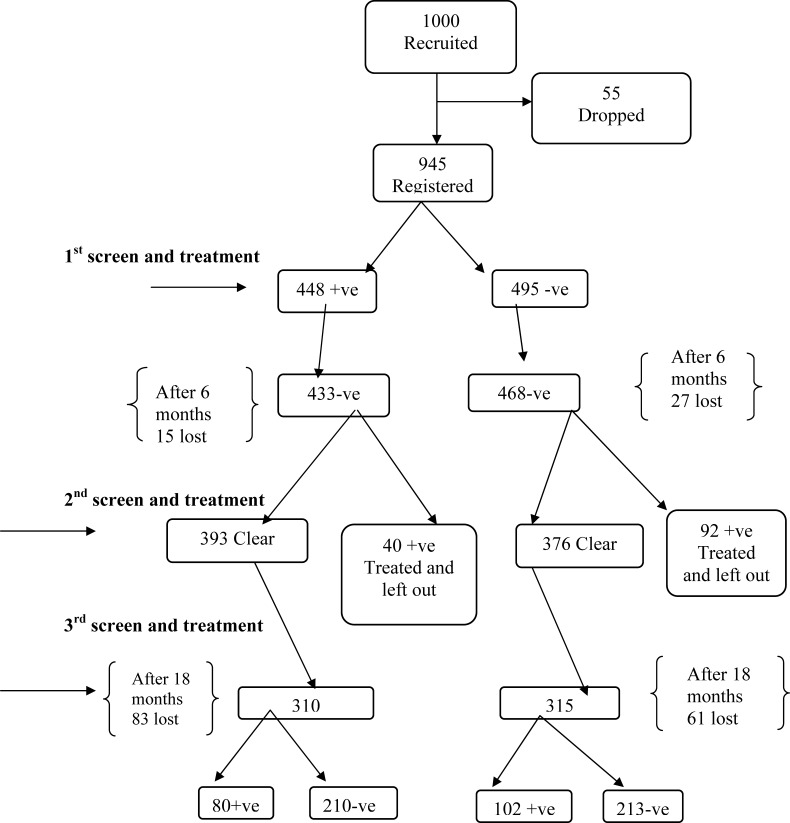
Flow chart showing the follow up of patients from first recruitment up to 18 months

A very small proportion (2.4%) of the 433 patients who were *S. mansoni* positive at the baseline survey continued to excrete eggs of *S. mansoni* in their faeces. Meanwhile among the 468 negative patients at the baseline (2.5%) of them were infected (Figure 1). Comparison of eggs intensity excreted by the 80 cohorts who were positive at base line, cleared their infections after six months but got re-infected twelve months later are shone in (Table 1).Meanwhile Comparison of Intensity of *S. mansoni* amongst 80 patients who were reinfected 18 months later and 102 patients who were *S. mansoni* negative but became *S. mansoni* positive 18 months later by Student's T-test did not find any significant difference This implies that the benefit of treatment with PZQ continues to be felt in the community even after 18 months (Table 2).

**Table 1:** Comparison of Intensity of *S. mansoni* amongst 80 cohorts who were reinfected 18 months but had cleared the infections after six months.

BASE LINE INFECTION OF THE 80 COHORTS				INFECTION AFTER 18 MONTHS OF SAME 80 COHORTS	
Age group	No of cohorts / % of infected before and after 18 months in age groups	Eggs excreted at base line	Geometric Mean count	Eggs excreted after 18 months	Geometric Mean count
5–10	9 (11.25)	2632	463.86	2139	133.77
11–20	47 (58.75)	18812	285.05	9680	151.97
21–30	13 (16.25)	9184	317.11	2662	151.74
31–40	7 (8.75)	2200	284.20	1924	156.43
≥ 41	4 (5.0)	1079	260.08	396	138.94
TOTAL	80 (100)	33897	285.05	16801	151.96

NB The Table 1: showed that there was 50.4 percent reduction in eggs excretion from the 80 cohorts studied at base line and 18 months later.

**Table 2:** Comparison of Intensity of *S. mansoni* amongst 80 patients who were reinfected 18 months later and 102 patients who were *S. mansoni* negative but became *S. mansoni* positive 18 months later.

Re-infection with *S. mansoni* 18 months later N=80				Infection with *S. mansoni* 18months amongst 102 patients who were initially *S.mansoni* negative			
Age group	N	Mean eggs	Std	N	Mean eggs	Std	T test (p-value)
5–10	9	133.77	202.8	23	79.4	119.9	0.555
11–20	47	151.97	173.2	47	60.9	85.6	0.031*
21–30	13	151.74	232.4	17	206.6	244.2	0.265
31–40	7	156.43	48.7	9	131	251.3	0.286
≥ 41	4	138.94	28.9	6	20	6.9	0.427
TOTAL	80	151.96	152.09	102	105.03	179.86	0.586

**Legend:** Std = standard deviation. N= 80 the total number individuals re- infected with *S. mansoni* after the second treatment and the 102 individuals who were negative initially but became infected at the end of the 18 months.

* The only age group which showed significant T test. ∑=Sum of mean

Comparing the difference between those who remained negative after treatment with those who were infected in the baseline study the force of clearance of PZQ showed significant difference (P= 0.001). Similarly the force of clearing the infection of PZQ after 18 was also significant (P=0.001). While the re-infection rate was significant (P=0.001) after six months there was no significant difference in the re-infection rate after 18 months (P= 0.766) (Table 2).

## Discussion

Several studies of infections and reinfection with *Schistosoma mansoni* after treatments with PZQ of residents living in endemic areas in the Tropics have been shown to lead to reduction of prevalence and intensity to re-infection after treatment. The re-infection prevalence and intensity have been shown never to equal before treatment level (Correa-Oliveira, et al., 2000). This study showed that there was reduction in the percentage of infection from {47.4% (448 out of 945) to 25.8% (80 out 310)} and the percentage reduction of the sum of eggs excretion was 50.4% in post PZQ therapy. This was 18 months after the initial treatment with PZQ 40mg/kg/wt. (Stelma et al., 1995; van.Liehout, et al., 1999).

We deliberately set a period of 18 months in total with the first six months for the assessment of the success of baseline treatment. We subsequently followed the cohorts up for twelve months to allow the study residents exposures to reinfection. This was to find out the level of resistance and susceptibility developed after treatment (Mott, 1982). Follow up studies to establish the force of reinfection after treatment with PZQ after short interval has been documented (Mott, 1982; Davis, 1993). Nevertheless this short term administration of treatment is uneconomical for most countries in the Tropic. Therefore, this study was to establish a treatment regiment for economical reasons for Kigungu and eventually elsewhere. Most schistosomiasis control Programmes in the Tropics are based on a six monthly single dose administration of PZQ to large communities. Repeated treatment to re-infected individuals is recommended depending on the degree of transmission in areas (Cioli, 2000). In spite of the low cost of PZQ, most counties in Africa are unable to procure adequate PZQ for mass chemotherapy. Study of this kind helps to establish treatment regiments for communities in schistosomiasis endemic areas in the tropical countries with meagre budget for helminth control programmes (Sleigh et al., 1985; Mott, 1982).

Mutapi in their study of changes in specific anti-egg antibody levels with PZQ followed their patients after 9 months (Mutapi, et al., 1998). In addition, Correa-Oliveira in their natural versus drug induced resistance in *S. mansoni* infections study followed their patients for five years at an interval of one year (Correa-Oliveira, et al., 2000). Our study demonstrating long term benefits of PZQ covered a period of 18 months. It is of importance to note here that Immunological responses due to dead *S.mansoni* adult worms and eggs antigens could be contributing to the resistance to the reinfections (Ana, et al.1995; Khalife, et al., 1986; Dunne, et al., 1992). Repeated treatment with PZQ within short intervals was also found to have no effect on the re-infection period in children in their first decade of life (Correa-Oliveira, et al., 2000). Nevertheless, the cure proportion of PZQ is greater if the treatment is repeated within a short period but the cost of the treatment remains relatively high (Correa-Oliveira, et al., 2000; Ongom and Bradley 1972).

On the other hand lower cure proportion of 18–39% was observed in very intense focus of *S. mansoni* infection in northern Senegal (Stelma et al., 1995; van.Leihout, et al., 1999). In our study, infections were lower for higher age groups ≥ 21 years indicating that there is relationship between *S. mansoni* infection and age (Tables 1 and 2). This is in line with phenomena commonly observed in endemic areas (Gryseels, 1994). Pre-treatment infections categorised according to the levels of intensity showed that 197 patients in Kigungu were in low intensity levels (1–100 epg), 145 of them were in the middle levels of intensity (101–400epg) and only 106 of them excreted high egg count greater than 401 epg (Sleigh et al., 1985). We observed that 213 cohorts remained *S. mansoni* negative through out the study. They confirmed to us that they have never been treated at any time for *S. mansoni*. The observation that 213 patients remained uninfected in all studies points out to an interesting situation in which some of the residents live all their lives in *S. mansoni* endemic areas but do not get the infection. This group of patients commonly known as endemic normal (putative resistance) was always stool negative for S*. mansoni* eggs. The putative resistant people of Kigungu have been found to be stool negative for *S. mansoni* before and after treatment (Gazzinelli, et al., 1985).

Intensities of infection influence manifestation of clinical schistosomiasis (N' Goran, 2003). From our records before treatment, majority of patients with multiple clinical symptoms such as abdominal pains, diarrhoea and blood in stool were positive for *S. mansoni* (Table 4). There were a few male patients with haematuria in this study but they were all negative for *S. haematobium*. Many studies showed that *S. mansoni* is the predominant species of schistosome in Uganda (Ongom and Bradley 1972; Odongo-Aginya and Mugisha, 1987). However, Schwertz 1951 and Cridland 1957 reported visceral schistosomiasis in Lango and Acholi (Schwert, 1951; Cridland, 1957). The few patients with ascites detected in this study were all positive for *S. mansoni*. Ascites is an indicator of chronic schistosomiasis with enlarged liver and spleen palpable (Davis, 1993; Ongom and Bradley 1972). Histories of blood vomiting were also recorded but they were not severe oesophageal varices bleeding linked to *S. mansoni*. The latter two clinical symptoms are characteristic of chronic schistosomiasis (Mott, 1982; Correa-Oliveira, et al., 2000). Age and pre-treatment intensity were the main host- parasite factors, which were significantly associated with *S. mansoni* low cure proportion in this study. This study also adds evidence of association between PZQ cure proportion, age and pre-treatment intensity. This observation stresses the need of two doses of PZQ within a standardised period to reduced morbidity due to heavy schistosome infections and avoids environmental contamination with *S.mansoni* eggs in endemic areas (N' Goran, 2003)

**Table 4:** Percentage of common clinical symptoms observed among the study participants.

Clinical symptoms %	Abdominal pain	Diarrhoea	Blood in stool	Blood in urine	Ascites	Blood vomiting	Asymptomatic
Before treatment	44.5(421*)	21.1(199*)	13.3(126*)	0.8(8*)	0.4(4*)	0.6(6*)	13.8 (130*)
After treatment	30.1(188*)	18.2(134*)	6.6(41*)	0	0	0	37(231)

**Legend:** Clinical symptoms observed* before and after treatment. Before treatment, *S. mansoni* positive patients had one or more of these clinical symptoms (not shown). Nevertheless after treatment most of the asymptomatic and some symptomatic patients became *S. mansoni* negative. There were reductions of major clinical symptoms while the minor symptoms disappeared after treatment. The percentage clinical symptoms was the ratio of observed symptoms to total number of the patients before or after treatment {(N/945*100); (N/625*100)}.

Our stool analysis was based on a single stool specimen from each patient. Nevertheless, three stool smears were prepared from each specimen to increase the accuracy of the egg count in each stool specimen (Ongom and Bradley 1972; Odongo-Aginya, et al., 1997). In most community-based studies, cure proportion have been estimated based on only one or two slides Kato/Katz reading usually derived from a single stool sample (Kathrine, et al., 1999). The multiple stool samples procedure is particularly relevant when the overall geometric mean egg count is low, because it increases the chances of estimating true cure proportion (Sleigh et al., 1985, Mott, 1982).

Our findings raise very high hope about the long term benefit of morbidity reduction initiated by praziquantel treatment. Nevertheless, since praziquantel is used widely in Africa especially in Uganda for large-scale treatment of schistosomiasis, it is relevant to monitor praziquantel effectiveness and the development of the parasites resistance to the drugs according to the set time frame of a particular endemic focus (Mott, 1982; Stelma et al., 1995; Correa-Oliveira, et al., 2000).

## Figures and Tables

**Table 3:** Cure rates and reinfection rates

Cure rates after 6 months		P value	OR (CI)
393/433	92/468	0.001	4.63 (3.53 – 6.06)
Cure rates after 18 months			
210/310	102/376	0.001	2.20 (1.87 – 3.34)
Re infection rate after 6 months			
40/432	92/469	0.001	0.47 (0.31 – 0.71)
Re infection rate after 18 months			
80/310	102/376	0.766	0.95 (0.68 – 1.34)

**Legend:** cure rate after 6 months and 18 months against force of re-infection in six months and 18 months after treatment In addition, the reductions of the clinical symptoms observed before and after treatment further demonstrate the evidence of long benefit of parazequantel in this community. In *S. mansoni* positive patients most patients had one or more clinical symptoms before treatment, (data not shown). Nevertheless after treatment, most of the asymptomatic and some with abdominal pains, diarrhoea and blood in stool became *S. mansoni* negative. These indicated that those persisting symptom could be of different causes other than those for *S. mansoni*. We detected mixed infection of *S.mansoni* with others intestinal parasites including soil transmitted helminths and protozoan infections. There were reductions of major clinical symptoms while the minor symptoms disappeared after treatment (Table 3).
